# Deadly Puppy Infection Caused by an MDR *Escherichia coli* O39 *bla*_CTX–M–15_, *bla*_CMY–2_, *bla*_DHA–1_, and *aac(6)-Ib-cr* – Positive in a Breeding Kennel in Central Italy

**DOI:** 10.3389/fmicb.2020.00584

**Published:** 2020-04-15

**Authors:** Vittoria Mattioni Marchetti, Ibrahim Bitar, Alessandra Mercato, Elisabetta Nucleo, Federica Marchesini, Marika Mancinelli, Paola Prati, Giada Simona Scarsi, Jaroslav Hrabak, Laura Pagani, Massimo Fabbi, Roberta Migliavacca

**Affiliations:** ^1^Unit of Microbiology and Clinical Microbiology, Department of Clinical-Surgical, Diagnostic and Pediatric Sciences, University of Pavia, Pavia, Italy; ^2^Biomedical Center, Faculty of Medicine in Pilsen, Charles University, Pilsen, Czechia; ^3^Department of Microbiology, Faculty of Medicine in Pilsen, Charles University, Pilsen, Czechia; ^4^Pavia Department, Istituto Zooprofilattico Sperimentale della Lombardia e dell’Emilia Romagna “Bruno Ubertini” (IZSLER), Pavia, Italy

**Keywords:** *E. coli*, DHA-1, CTX-M-15, CMY-2, whole genome sequencing, plasmids

## Abstract

Antimicrobial consumption in veterinary medicine has led to the spread of multi drug-resistance in clinically important bacteria, with the companion animals and their environment involved as emerging reservoirs. While CTX-M-15 and CMY-2 acquired β-lactamases have been widely detected in the bacterial population of companion and breeding animals in European area, DHA-1 enzymes have been rarely reported in veterinary medicine. The aim of the study was to characterize the *Escherichia coli* associated with mortality of a litter of Bulldog puppies in a breeding kennel located in Pesaro area, Central Italy. The *E. coli* strains O39 serotype were resistant to 3rd/4th generation cephalosporins, chloramphenicol, aminoglycosides, trimethoprim-sulfamethoxazole, and ciprofloxacin, retaining susceptibility to carbapenems, colistin, fosfomycin, and levofloxacin (by Microscan Autoscan4, EUCAST clinical breakpoints). Pulse field gel electrophoreses (PFGE-XbaI) on five *E. coli* strains revealed the presence of a single profile. Whole genome sequencing (WGS) analysis revealed a complex resistome, harboring *bla*_TEM–1b_, *bla*_CTX–M–15_, *bla*_OXA–1_, *aph(6)-Ib*, *aac(6′)Ib-cr*, *aac(3)-Ila*, *aph(6)-Id*, *aadA1*, *qnrB1*, *sul2*, *catA1*, *catB3*, *tetA*, and *dfrA14* genes located on a 302597 bp IncHI2/HI2A plasmid. Moreover, *bla*_DHA–1_, *qnrB4*, *mph(A)*, *sul1*, and *dfrA17* determinants were carried on an 83,429 bp IncFII plasmid. A *bla*_CMY–2_ determinant was carried on a 90,249 bp IncI1 plasmid. Two IncX1 and IncX4 plasmids without antimicrobial resistance genes were also detected. The presence of *lpfA*, *iss*, *astA*, and *gad* virulence factors was highlighted. This is the first report in Italy on an invasive infection in eight 2-weeks old dogs caused by the same MDR *E. coli* O39 *bla*_CTX–M–15_, *bla*_CMY–2_, *bla*_DHA–1_, and *aac(6′)-Ib-cr* positive strain. The above MDR *E. coli* clone caused the death of the entire litter, despite amoxicillin-clavulanate and enrofloxacin administration. The tank for storage of the water used to prepare the milk-based meal for the litter was the suspected reservoir.

## Introduction

Antimicrobial drugs are extensively used in veterinary medicine for therapy, prophylaxis and metaphylaxis (Marshall and Levy, 2011). The amount of antibiotics consumption in livestock and companion animals is almost double the one used in humans, even though this ratio decreased in the recent years (The European Centre for Disease Prevention Control [ECDC] et al., 2017; King et al., 2018). Nevertheless, the spread of antimicrobial-resistant bacteria between animals and humans is leading to further antimicrobial resistance (AMR) dissemination (Guardabassi et al., 2004; Harada et al., 2011; Chirila et al., 2017). Acquired AMR mechanisms have been found in *Escherichia coli* collected from veterinary medicine, such as ESBLs, conferring resistance to aminopenicillins, 3rd/4th generation cephalosporins, and monobactam, and Ambler group C (AmpC) β-lactamases, hydrolyzing penicillins and broad spectrum cephalosporins (Marshall and Levy, 2011). Furthermore, multidrug-resistant (MDR) *Enterobacterales* isolates have been reported extensively in veterinary practices from pets and farm animals ([Bibr B32][Bibr B27]). MDR *E. coli* isolates bearing plasmid-mediated AMR, have been increasingly isolated from both animals and the environment (Manaia et al., 2016; Wang et al., 2017).

CMY-2 cephalosporinases are the most important AmpCs enzymes detected in bacteria isolated from human, animal, and environmental specimens (Yilmaz et al., 2013; Pehlivanlar et al., 2015; Dolejska and Papagiannitsis, 2018). The *bla*_DHA__–type_ genes have been rarely detected in veterinary medicine (Belas et al., 2014), mainly in *Klebsiella pneumoniae* and *E. coli* from companion animals (Hidalgo et al., 2013). Plasmid-mediated ESBLs/AmpC have been widely reported in bacterial strains isolated from pets (Liu et al., 2016; Zogg et al., 2018) and the *bla* resistance determinants dissemination limits the treatment options. Moreover, the co-presence of *aac(6′)-Ib-cr* and ESBL/AmpC – encoding genes has been recently detected in companion pets (Zogg et al., 2018) and breeding animals (Jones-Dias et al., 2016; Röderova et al., 2017; Yang et al., 2018). The aim of this study was to characterize the *E. coli* associated with mortality of a litter of Bulldog puppies in a breeding kennel in Pesaro area, Central Italy, which lead to the death of the eight puppies (entire litter).

## Materials and Methods

### Bacterial Isolation

A total of five *E. coli* strains (*E. coli*1feg and *E. coli*2feg from liver biopsies, and *E. coli*6feg, *E. coli*7feg, and *E. coli*8feg from gut biopsies) were collected on September 8th, 2017, from gut and liver biopsy specimens of five 2-week old Bulldog puppies. The puppies lived in a dog breeding kennel located in Tavullia (PU) and belonged to an eight-puppy litter. At the beginning of September, the entire litter had hemorrhagic enteritis with renal involvement and cerebral vessels congestion. Puppies were treated for 3 days with amoxicillin plus clavulanic acid, then switched to enrofloxacin. However, the eight puppies died after ineffective treatment strategies.

### Identification, Antimicrobial Susceptibility Testing and Serotyping

The strain species identification was confirmed using matrix-assisted laser desorption ionization-time of flight mass spectrometry (MALDI-TOF MS) using MALDI Biotyper software (Bruker Daltonics, Bremen, Germany). Species identification and antimicrobial susceptibility was then assessed by MicroScan AutoScan-4 (Beckman-Coulter) and confirmed by broth microdilution. MICs were interpreted according to EUCAST, 2019 clinical breakpoints^1^. Serotyping was performed by hot tube agglutination with specific sera as described elsewhere (Qrskov and Orskov, 1984; Ewing, 1986).

### Antimicrobial Resistance Gene Investigations

Check-MDR CT103XL (checkpoint) microarray and/or polymerase chain reaction (PCR) and sequencing were used for AMR genes investigation as described elsewhere (Bogaerts et al., 2016). The primers’ sequences and PCR conditions used for the detection of the related genes were run as previously described (Pagani et al., 2003; Weill et al., 2004; Robicsek et al., 2006; Liu and Liu, 2016; Peymani et al., 2016).

### Conjugation Experiments

Conjugation experiments for the five isolates (*E.coli*1feg, *E.coli*2feg, *E.coli*6feg, *E.coli*7feg, and *E.coli*8feg) were performed using rifampin resistant *E. coli* K12 J53 (met−, pro−, lac+) streptomycin resistant *E. coli* K12 J62 (pro−, his−, trp−, lac, Smr) as recipients, selecting transconjugants on 100 μg/ml rifampicin plus 8 μg/ml cefotaxime and on 100 μg/ml streptomycin plus 8 μg/ml cefotaxime as described elsewhere (Piazza et al., 2015; Caltagirone et al., 2017). The presence of *bla*_DHA_, *bla*_CTX–M__–type_, and *bla*_CMY–2_ was confirmed by PCR and the plasmid replicon presence was confirmed using the PBRT kit (Diatheva, Fano, Italy).

### Pulse Field Gel Electrophoresis

For pulse field gel electrophoresis (PFGE), the five *E. coli* isolates were grown on MacConkey agar at 37°C for 24 h. The plug preparation and PFGE were performed according to the standardized PulseNet International protocol for *E. coli* O157:H7, *E. coli* non-O157, *Salmonella* serotypes, *Shigella sonnei*, and *Shigella flexneri*^2^. The macrorestriction digestion was performed applying *Xba*I (40 U/sample; Promega Corporation; Madison, WI, United States) at 37°C for 19 h. Fragments were separated in a 1% (w/v) Pulsed Field Certified Agarose gel (Bio-Rad, Hercules, CA, United States) in a 0.5× TBE buffer on a CHEF DRII system (Bio-Rad, Hercules, CA, United States) at 14°C at 6 V/cm for 25 h with an initial pulse time of 0.5 s and a final pulse time of 30 s. Lambda 48.5 kb ladder (New England BioLabs, Beverly, MA, United States) was used as molecular size marker. The gel was stained with ethidium bromide (Sigma-Aldrich, Vienna, Austria), digitally photographed with Gel Doc 2000 (Bio-Rad Laboratories, Inc.) and normalized as TIFF images. Dendrogram of strain relatedness was created with Fingerprinting II version 3.0 software (Bio-Rad) using UPGMA. The Dice correlation coefficient was used with a 1.0% position tolerance to analyze the similarities of the banding patterns. The restriction patterns of the genomic DNA from the isolates were analyzed and interpreted according to the criteria described previously (Tenover et al., 1995).

### Plasmid Size

The size of the plasmids that carried *bla*_DHA__–1_, *bla*_CTX–M–15_, and *bla*_CMY–2_, genes was detected by PFGE of total DNA digested with S1 nuclease (Promega, Madison, WI, United States; Barton et al., 1995). Then the DNA was transferred to a BrightStar-Plus positively charged nylon membrane (Applied Biosystems, Foster City, CA, United States) and hybridized with *bla*_CTX–M–15_, *bla*_DHA–1_, and *bla*_*CMY*−2_ probes labeled with digoxigenin as described elsewhere (Mavrodi et al., 2009).

### Whole Genome Sequencing, Annotation, and Plasmid Analysis

The MICs, AMR profile and dendrogram from PFGE suggested that the five isolates were indistinguishable; hence, the genomic DNA of two *E. coli* strains (chosen as representatives, for comparison purposes) was extracted using NucleoSpin Microbial DNA Kit (Macherey-Nagel, Germany). Sequel I platform (Pacific Biosciences, CA, United States) was used for sequencing. Library preparation was done following the microbial multiplexing protocol according to the manufacturer’s instructions for sheared DNA. Shearing was performed using g-tubes (Covaris, United States), and no size selection was done during the library preparations. HGAP4 was used to perform the assemblies of the genomes with minimum seed coverage of 30. ResFInder 3.2^3^ (Zankari et al., 2012), PlasmidFInder^4^ (Carattoli et al., 2014), VirulenceFinder 2.0^5^ (Joensen et al., 2014) ISfinder database^6^, MLST 2.0^7^ (Larsen et al., 2012), and CHTyper 1.0^8^ (Camacho et al., 2009) were utilized to detect resistance genes, plasmid replicon type, virulence genes, mobile elements, Sequence type (ST) and FimH/FumC type, respectively. Open reading frames (ORF) were predicted using RAST 2.0 (Brettin et al., 2015) with default parameters combined with BLASTP/BLASTN. Comparative genome alignments were performed using the Mauve (version 2.3.1). Gene organization and diagrams were sketched using Inkscape 0.92.4^9^.

### Nucleotide Sequence Accession Numbers

The nucleotide sequences of pIV_IncI1, pIV_IncHI2_CTX_M_15, and pIV_IncFII_DHA plasmids were deposited in the GenBank under the accession numbers MN540570, MN540571, and MN537908, respectively.

## Results

All the isolates revealed resistance to ampicillin, piperacillin, third generation cephalosporins, aztreonam, ciprofloxacin, gentamicin, and moxifloxacin, while susceptibility to carbapenems, colistin, fosfomycin, and levofloxacin (The European Committee on Anti-microbial Susceptibility Testing – EUCAST, 2019; breakpoints) was retained. Moreover, the conjugation experiment showed the transferability of all the AMR determinants to the transconjugants (Supplementary Table S1). The collected isolates showed identical profile by PFGE, pointing out that a single clone was involved in the infection (Supplementary Figure S1). Therefore, based on the similar PFGE pattern, MICs and resistance profile, whole genome sequencing (WGS) was performed on two representatives of the five strains. WGS revealed that the above isolates were almost identical for genomic sequence (99% sequence identity); moreover, the plasmids sequences showed 100% of sequence similarity and coverage. The highest sequencing coverage in depth was obtained from the *E. coli*4feg plasmids (504×), thus used as a reference.

**FIGURE 1:**
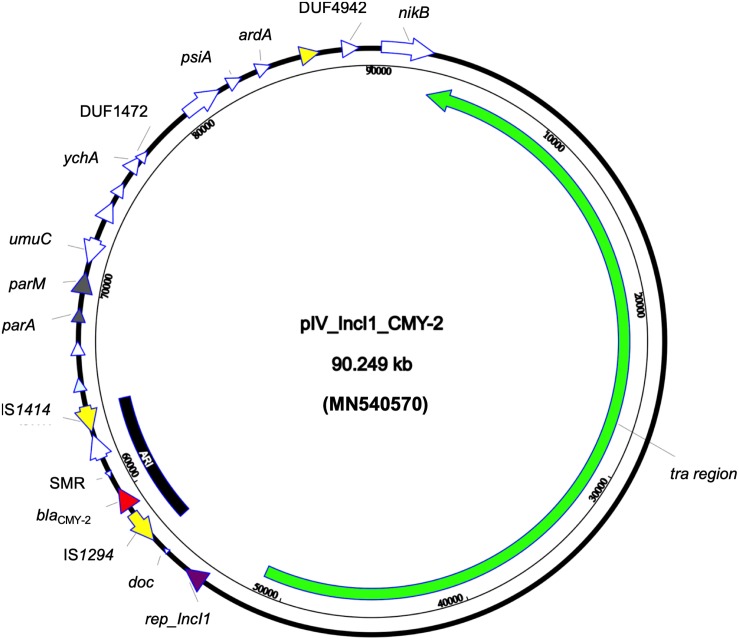
Circular map of pIV_IncI1_CMY-2 plasmid. Green arrow represent conjugal transfer system of the plasmid, red arrow represents *bla*_CMY–2_, yellow arrows represent mobile elements, white arrows represent hypothetical proteins, purple arrow represents the replication protein rep_IncI1, and gray arrows represent plasmid partitioning proteins. Furthermore, the black-arced box represents the plasmid antimicrobial resistance island (ARI).

Whole genome sequencing revealed that the strain belonged to ST ST58/ST186 (according to Oxford and Pasteur scheme, respectively) which belongs to the clonal complex (CC) ST155, serotype O39:H21 and revealed the presence of FumC4/FimH32 fimbriae types. Furthermore, the strain carried a set of different virulence genes: two copies of *gad*, *astA*, *iss*, and *lpfA* coding for glutamate decarboxylase, heat stable toxin, increased serum survival protein, and long polar fimbriae, respectively. The beta-lactamase and *aac(6′)-Ib-cr* genes were transferable through conjugation; both the donor and transconjugants AMR gene content was confirmed through PCR. The *E. coli*4feg harbored five plasmids. No AMR nor virulence genes was detected in IncX1 and IncX4 -type plasmids. An additional relatively large (90,249 bp) IncI1 plasmid (pIV_IncI1_CMY-2) showed a high sequence similarity to a plasmid identified in the United States in 2017 (CP024854.1; 99.99% sequence identity and 98% sequence coverage). The pIV_IncI1_CMY-2 backbone was highly conserved, having genes for replication (*repZ*), conjugative transfer system (*tra*, *pil* genes), and maintenance (*parA*, *parM*). Moreover, the above plasmid carried a 7,446 bp antimicrobial resistance island (ARI) harboring only a *bla*_CMY–2_ determinant and a multidrug efflux resistance gene, flanked by two insertion sequences (IS) IS*1414* and IS*1294* in the same orientation (Figure 1).

**FIGURE 2:**
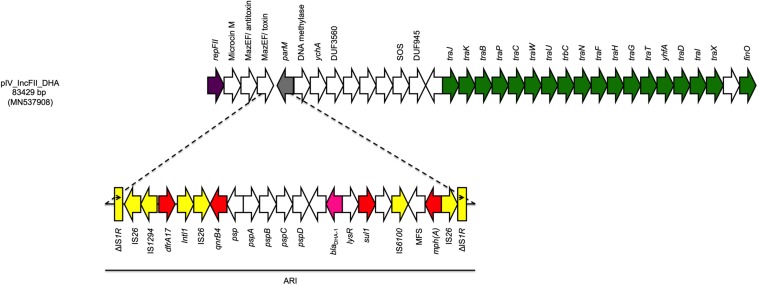
Linear map of pIV_IncFII_DHA. Arrows show the direction of transcription of ORFs while rectangles show truncated ORFs. Replicons, partitioning genes, mobile elements, conjugal transfer genes, antibiotic resistance, *bla*_DHA–1_, and other remaining genes are designated by violet, gray, yellow, green, red, pink, and white, respectively. Antimicrobial resistance island (ARI) is marked by a horizontal black line.

The fourth plasmid (pIV_IncFII_DHA) was 83,429 bp in size and belonged to the IncFII plasmid family. pIV_IncFII_DHA showed high score with both the p133355_SW_C4_Cam-1 plasmid (81,724 bp) reported in 2019 in a *Citrobacter amalonaticus* collected from a human stool specimen in Switzerland (CP041363.1; 99.64% sequence identity, 97% sequence coverage), and with the pUB_DHA-1 plasmid (81,754 bp), present in an *E. coli* strain from a human urine sample in United Kingdom (MK048477; 99.55% sequence identity, 97% query coverage) (Findlay et al., 2019). The plasmid backbone carried regions responsible for replication (*repA*), conjugative transfer system (*tra*, *yhfA, finO* genes), maintenance (*parM*) and toxin-antitoxin (TA) systems (*mazEF/mazEF).* Moreover, the plasmid carried a 22,425 bp ARI harboring genes coding for cephalosporin resistance (*bla*_DHA–1_), sulphonamide resistance (*sul1*), trimethoprim resistance (*dfrA17*), macrolide resistance (*mph(A)*), and quinolone resistance (*qnrB4*). The ARI was characterized by a high number of IS, located between the AMR genes. Interestingly, such IS were flanked by two IS*26* with opposite orientation, followed by truncated IS*1R* on both ends in the same direction (Figure 2).

**FIGURE 3:**
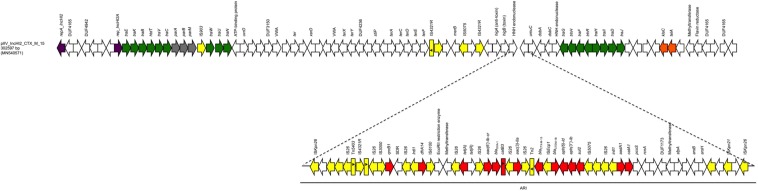
Linear map of pIV_IncHI2_CTX_M_15. Arrows show the direction of transcription of ORFs while rectangles show truncated ORFs. Replicons, partitioning genes, mobile elements, conjugal transfer genes, antibiotic resistance, stability genes, and other remaining genes are designated by violet, gray, yellow, green, red, orange, and white, respectively. Antimicrobial resistance island (ARI) is marked by a horizontal black line.

The last plasmid (pIV_IncHI2_CTX_M_15) was the largest, with a size of 302,597 bp; it belonged to the IncHI2 plasmid family. The pIV_IncHI2_CTX_M_15 exhibited high sequence similarity scores both with a 300,375 bp plasmid, carried by a *Salmonella typhimurium* strain detected in a Kenyan patient in 2015 (LN794248.1; Kariuki et al., 2015) (100% sequence identity, 99% sequence coverage), and with the 309,608 bp pEc21617-310 plasmid carried by an *E. coli* isolated from a human urine specimen in Taiwan in 2019 (MG878867.1; 99.99% sequence identity, 96% sequence coverage). The pIV_IncHI2_CTX_M_15 backbone carried genes responsible for replication (*repA, repB*), conjugative transfer system (*tra*, *trhR, hldT* genes), stability (*telA*, *klaC*), maintenance (*parM, parA, parB*), arsenic resistance system (*arsB, arsH*), tellurium resistance genes (*terA, terC, terD, terE, terF, terX*), and TA systems (*higB/higA).* Furthermore, the plasmid carried a 91,179 bp ARI which harbored genes coding for β-lactam resistance (*bla*_OXA–1_, *bla*_CTX–M–15_, and *bla*_TEM–1B_), fluoroquinolone/aminoglycoside resistance [*aac(6′)-lb-cr*, *aac(3)-IIa*, *aph(6)-ld*, *aph(3″)-lb*, *aadA1*], sulphonamide resistance (*sul2*), phenicol resistance (*catB3, catA1*), trimethoprim resistance (*dfrA14*), tetracycline resistance [*tet(A)]*, and quinolone resistance (*qnrB1*). In addition, between the resistance genes and the ends, the ARI region carried a large number of IS elements, with IS*26* as the most prevalent. Finally, the ARI was flanked by two IS*Kpn26*, in same orientation (Figure 3).

## Discussion

Here, we describe a case of a severe deadly infection caused by a MDR *E. coli* clone in a bulldog breeding kennel, in Italy. Bacterial isolates in pets, breeding and food-chain animals act as an important factor in the zoonotic transmission of fluoroquinolone, third and fourth generation cephalosporin, carbapenem and recently colistin antibiotic resistance genes (Dolejska and Papagiannitsis, 2018). Nevertheless, the co-presence of *bla*_CTX–M–15_, *bla*_CMY–2_, *bla*_DHA–1_, and *aac(6′)-Ib-cr* genes in the same toxigenic strain of animal origin is an alarming concern not reported previously. Moreover, rare cases of *E. coli bla*_DHA–1_ positive in veterinary medicine are reported (Belas et al., 2014).

*Escherichia coli* O39:H21 has been rarely reported globally except for some reports relating it to be isolated from animals (Constantiniu, 2002). The CC ST155 has been identified in human, animal, and environmental samples as reported elsewhere (McKinnon et al., 2018). Furthermore, most of the reports indicate plasmid mediated production of CTX-M-15 within this ST (Chen et al., 2016; Mohsin et al., 2017; Blanco et al., 2019).

Moreover, the genetic structure of the three out of five described plasmids is well conserved, showing high similarity with globally reported plasmid sequences from bacteria derived from both human and animal/environmental settings. This suggests that the global and interspecies spread of plasmids like pIV_IncHI2_CTX_M_15 and pIV_IncFII_DHA plays a central role in MDR spread (Rozwandowicz et al., 2018). pIV_IncI1_CMY-2 carried a relatively small ARI, yet the plasmid backbone showed a long conjugal transfer system which accounted for almost half of the plasmid size. Interestingly, the presence of complete IS elements at the ends of ARI suggests the ability to acquire additional gene cassettes of other ISs by integration/recombination events and also suggests the hypothesis of this ARI dissemination through auto-insertion in similar replicon plasmids as reported elsewhere (Bitar et al., 2019b).

Despite having a conserved plasmid backbone, the ARI of pIV_IncFII_DHA was larger than the one found in p133355_SW_C4_Cam-1 plasmid. The presence of a high number of ISs suggests that this region contains hotspots, where IS flanked ARIs can be integrated. Moreover, the sequence array suggests that this region initially was a composite transposon flanked by two IS*26* in opposite direction. This also suggests that an insertion of the complex transposon in an IS*1R* gene occurred either directly or through recombination as reported in similar cases elsewhere (Bitar et al., 2019a) supported by the fact that two truncated IS*1R* are flanking the transposon, with one corresponding to the beginning of the gene (1–473 out of 768 bp) and the other one to the end of the gene (291–768 out of 768 bp). Finally, the inverted repeats of IS*1R* were detected, with IRL found at one end and the IRR on the other (Figure 2), and no direct repeats were found at the insertion site of the complex transposon.

The plasmid pIV_IncHI2_CTX_M_15 backbone was well conserved in its most parts, except for: (i) an IS*903* in the *tra* region next to the plasmid partitioning genes and (ii) an inserted small region flanked by two IS*4321R*, one of which truncated due to the presence of another transposase located in close proximity. This region carried few genes coding for hypothetical proteins, and a *merB* gene coding for an organomercurial lyase. The ARI harboring an array of AMR determinants, also carried 23 genes corresponding to transposases, integrons and ISs. The presence of ISs together with the described mosaic structure confirms the high predisposition of this region to acquire exogenous elements, as it occurred for the already inserted 14 AMR genes. Moreover, the IS*Kpn26* flanking the region could act as a complex transposon mediated by these two ISs (Figure 3); however, DR were found in the potential insertion site as described elsewhere (Bitar et al., 2019a).

The source of the infectious disease of the entire litter remains unknown; however, the breeding kennel had a previous history of *E. coli* infections; in fact, during pregnancy, the puppies’ mother was affected by a *Streptococcus pyogenes* and *Mycoplasma* spp. infection; such pathogens were eradicated using a ciprofloxacin and cefaclor combined therapy. At the time of the disease emergence, the possible involvement of an *E. coli* strain had never been considered. During the same period, a strong drought had struck in the Pesaro province. Worryingly, at that time, the villagers got water for every-day use from the same cistern that kennel’s owner used for the powdered milk preparation for the puppies. We hypothesized that the cistern water could be a source of the pathogen, although any *E. coli* clonally related to the one here described was not detected as cause of infection in the human population of this geographic area. Other possible sources could be the kennel environment, other dogs in the kennel or the caretakers themselves. Nevertheless, no other similar infection has been reported neither in the dogs in the kennel nor the caretakers working there. In summary, for a better comprehension of the “spread routes” of clinically important MDR clones, and in accordance with the universally shared “One Health approach,” it appears essential to enhance epidemiological surveillance in veterinary medicine and in environmental setting, in Italy.

## Data Availability Statement

The datasets generated for this study can be found in GenBank under the accession numbers: MN540570, MN540571, and MN537908.

## Ethical Approval

Ethical approval was not required since the research was conducted on strains isolated from the biopsies performed on the animal subjects after their death in the attempt to explain the cause of death. The strains were isolated from the biopsies which was sent to us by the owner of the kennel.

## Author Contributions

IB, VM, and RM played an important role in interpreting the results and writing the manuscript. AM, EN, FM, MM, PP, GS, JH, LP, and MF helped to acquire the data. IB and VM carried out the experimental work. IB supervised the experiments and revised the final manuscript, which was approved by all authors.

## Conflict of Interest

The authors declare that the research was conducted in the absence of any commercial or financial relationships that could be construed as a potential conflict of interest.

## Footnotes

1 http://www.eucast.org

2 https://www.cdc.gov/pulsenet/pdf/ecoli-shigella-salmonella-pfge-protocol-508c.pdf

3 https://cge.cbs.dtu.dk/services/ResFinder/

4 https://cge.cbs.dtu.dk/services/PlasmidFinder/

5 https://cge.cbs.dtu.dk/services/VirulenceFinder/

6 www-is.biotoul.fr/

7 https://cge.cbs.dtu.dk/services/MLST/

8 https://cge.cbs.dtu.dk/services/chtyper/

9 https://inkscape.org/
