# Supplementary material for: Deadly Puppy Infection Caused by an MDR Escherichia coli O39 blaCTX–M–15, blaCMY–2, blaDHA–1, and aac(6)-Ib-cr – Positive in a Breeding Kennel in Central Italy
Source: Front Microbiol. 2020 Apr 15;11:584. doi: 10.3389/fmicb.2020.00584 (PMC7174561; doi:10.3389/fmicb.2020.00584)
Supplement: Supplementary file 1 [file Data_Sheet_1.docx]

Table S1:

Antimicrobial susceptibility profile of conjugation recipient strain J53, donor strain E. coli4feg and the transconjugants J53_E. coli4feg

| **Isolate** | **MIC (mg/L)** |  |  |  |  |  |  |  |  |  |  |  |  |  |
| --- | --- | --- | --- | --- | --- | --- | --- | --- | --- | --- | --- | --- | --- | --- |
|  | **AMP** | **PIP** | **CAZ** | **CTX** | **ATM** | **CIP** | **GEN** | **MXF** | **MEM** | **IMP** | **ETP** | **CST** | **FOF** | **LVX** |
| **E. coli 4feg** | >8 | >16 | >8 | >16 | >16 | >1 | >4 | >1 | 0.5 | 1 | 0.5 | 1 | 32 | 1 |
| **J53_E coli4feg** | >8 | >16 | >8 | >16 | >16 | >1 | >4 | >1 | 0.5 | 1 | 0.5 | 1 | 32 | 1 |

MIC, minimum inhibitory concentration; AMP, ampicillin; PIP, piperacillin; CAZ, ceftazidime; CTX, cefotaxime; ATM, aztreonam; CIP, ciprofloxacin; GEN, gentamicin; MXF, moxifloxacin; MEM, meropenem; IMP, imipenem; ETP, ertapenem; CST, colistin; FOF, fosfomycin; LVX, levofloxacin


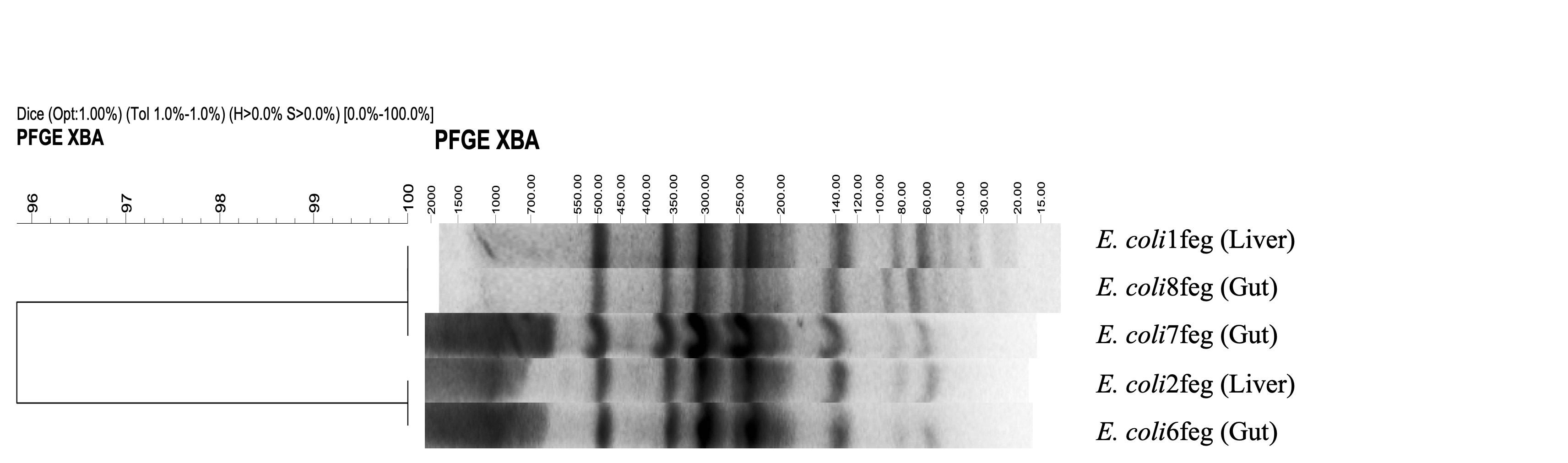


Figure S1: Cluster analysis of the ESBL-producing *E. coli* isolates (Dice coefficient: optimization 1,0% and tolerance 1,0%). The scale bar at the top (left) indicates similarity coefficient (%).
